# Novel Biallelic *INTS1* Variants May Expand the Phenotypic Spectrum of *INTS1*-Related Disorders—Case Report and Literature Review

**DOI:** 10.3390/genes16091081

**Published:** 2025-09-15

**Authors:** Aleksandra Wnuk-Kłosińska, Anna Sowińska-Seidler, Michał Piechota, Aleksander Jamsheer

**Affiliations:** 1 Department of Medical Genetics, Poznan University of Medical Sciences, Rokietnicka 8 Street, 60-806 Poznan, Poland; 2 Doctoral School, Poznan University of Medical Sciences, Bukowska 70 Street, 60-812 Poznan, Poland; 3 Diagnostyka GENESIS Sp. z o.o., Dąbrowskiego 77A, 60-529 Poznan, Poland

**Keywords:** whole-exome sequencing, *INTS1*, multiple congenital anomalies, dysmorphic features, developmental delay, autism spectrum disorder

## Abstract

**Background/Objectives:** Neurodevelopmental disorders (NDDs) are genetically heterogeneous conditions with a complex molecular etiology involving numerous genes. Biallelic pathogenic variants in *INTS1* cause a rare autosomal recessive NDD characterized by congenital cataracts, growth retardation, facial dysmorphism, and global developmental delay. To date, the clinical description of this disorder has been based solely on individual case reports, and its phenotypic spectrum remains incompletely defined. **Methods:** A 9-year-old female proband was evaluated for developmental delay, multiple congenital anomalies, and distinctive craniofacial features. Whole-exome sequencing (WES) was performed, followed by Sanger validation and segregation analysis. Variant pathogenicity was assessed using in silico prediction tools and 3D protein structural modeling. **Results:** Whole-exome sequencing identified two novel compound heterozygous missense variants in *INTS1*, c.1145G>A (p.Arg382Gln) and c.1195G>A (p.Gly399Ser), both located in exon 9. Segregation analysis showed that c.1145G>A was inherited from the father and c.1195G>A from the mother, and both variants are extremely rare in population databases. **Conclusions:** We report a patient carrying novel biallelic *INTS1* variants, whose clinical presentation differs from previously reported cases, including those with milder phenotypes characterized by preserved speech development and absence of intellectual disability. This observation broadens the clinical spectrum of *INTS1*-related disease and underscores its phenotypic heterogeneity.

## 1. Introduction

Neurodevelopmental disorders (NDDs) and congenital anomalies are clinically and genetically heterogeneous conditions with complex molecular etiology. Advances in next-generation sequencing (NGS) have facilitated the identification of an increasing number of genes and variants implicated in these disorders, enabling more precise molecular diagnoses and expanding our understanding of their phenotypic spectrum [1,2,3].

In 2017, Oegema et al. first described patients with biallelic pathogenic variants in the *INTS1* (Integrator Complex Subunit 1) gene, which encodes the largest subunit of the Integrator complex [4]. The affected individuals presented with congenital cataracts, developmental delay, and dysmorphic features. The Integrator complex (INT) is a phylogenetically conserved multiprotein assembly composed of at least 14 subunits that associates with the C-terminal domain of RNA polymerase II [5,6]. Initially identified as a key factor in the 3′-end processing of U-rich small nuclear RNAs (snRNAs), which are essential components of the spliceosome [7,8], it has since been implicated in a broader range of functions, including the regulation of transcriptional pausing and the processing of enhancer RNAs.

Since the initial report by Oegema et al. [4], additional cases have confirmed the association of biallelic *INTS1* variants with a rare autosomal recessive neurodevelopmental disorder [9,10,11], now listed in the OMIM database as Neurodevelopmental disorder with cataracts, poor growth, and dysmorphic facies (NDCAGF; #620148), although its phenotypic spectrum still appears to be incompletely defined.

Here, we report a patient carrying previously unreported biallelic *INTS1* variants, exhibiting a phenotype distinct from previously described cases. This observation expands the clinical spectrum of *INTS1*-related disorder and highlights its phenotypic heterogeneity.

## 2. Materials and Methods

Peripheral blood samples were collected from the proband and her unaffected parents. Genomic DNA (gDNA) was extracted from peripheral blood leukocytes using a MagCore HF16 Automated Nucleic Acid Extractor (RBC Bioscience, New Taipei City, Taiwan). DNA quantity and quality were assessed using an Agilent 2200 TapeStation System (Agilent Technologies, Santa Clara, CA, USA) and a Qubit fluorometer (Thermo Fisher Scientific, Waltham, MA, USA). The proband was clinically assessed by a clinical geneticist, including a review of her medical records and family history. Written informed consent was obtained from the parents prior to genetic testing, as well as for publication of the case report and clinical photographs.

The molecular diagnostic algorithm began with array comparative genomic hybridization (aCGH)—format 60K (Agilent Technologies, Santa Clara, CA, USA)—which was applied in order to detect copy number variants (CNVs) in the proband. The assay was performed according to the standard protocols provided by the manufacturers, using a non-commercial female control DNA. The results were analyzed using Agilent CytoGenomics 5.0.2.5 software (Agilent Technologies, Santa Clara, CA, USA). The aCGH results did not reveal any CNVs that could contribute to the patient’s abnormal phenotype. Accordingly, whole-exome sequencing (WES) was applied.

### 2.1. Whole-Exome Sequencing

WES was carried out by a certified commercial laboratory on a sample obtained from the patient. The coding and flanking intronic regions were enriched using a custom in-solution hybridization-based whole-exome capture device (Twist Bioscience, San Francisco, CA, USA) and sequenced on an Illumina NovaSeq platform (Illumina, San Diego, CA, USA). Bioinformatics processing involved demultiplexing using Illumina bcl2fastq2, adapter trimming with Skewer, and alignment to the hg19 human genome reference using a Burrows–Wheeler Aligner (BWA). PCR duplicates and ambiguously mapping reads were removed. Variant calling was conducted targeting single-nucleotide variants (SNVs) and small insertions/deletions (indels), followed by annotation using internal and external databases.

Variant filtering was limited to SNVs and indels in coding regions and ±8 bp of adjacent introns with a minor allele frequency (MAF) < 1.5%. Known pathogenic variants were also assessed within ±30 bp and up to 5% MAF based on HGMD, gnomAD, and an in-house database. Variants were classified in accordance with the ACMG guidelines [12]. The in silico prediction tools used included MutationTaster 2021, SIFT v5.2.2, PROVEAN v1.1, LRT, fathmm v2.3, fathmm-MKL v2.3, and Mutation Assessor r3. Splicing effects were evaluated using SpliceAI v1.3.1 with the pathogenicity threshold set at ≥0.8. Only predictions with sufficient consensus across tools were considered for interpretation. All variants were named following HGVS nomenclature. The assay fulfilled internal quality standards, including the requirement for ≥99.9% of targeted bases to be covered by at least 30 high-quality reads.

### 2.2. Sanger Sequencing

PCR followed by Sanger sequencing was performed in order to confirm the WES results in the proband and to test the presence of the identified variants in her parents. Each PCR was conducted in a total volume of 10 μL of the reaction mixture containing 5 µL of FailSafe™ 2X PCR PreMix J (Biosearch Technologies, Hoddesdon, UK); 0.5 μL of forward (5′-GAGCGTGGAGGAGTATGTCC-3′) and reverse (5′-AGGACCTGCATGTTGTTCG-3′) primer each (10 μmol/l each); 0.1 μL of Taq DNA polymerase (GenScript, NJ, USA); 0.5 μL of gDNA (150 ng/μL); and 3.4 μL of PCR-grade water. The reaction conditions were as follows: an initial denaturation step at 95 °C for 3 min followed by 35 cycles of denaturation at 94 °C for 30 s, annealing at 60 °C for 30 s, elongation at 72 °C for 45 s, and final elongation at 72 °C for 10 min. The PCR products were purified using a mixture of Exonuclease I (Exo I) with Shrimp Alkaline Phosphatase (SAP) (Thermo Fisher Scientific, Waltham, MA, USA) and sequenced using dye-terminator chemistry (kit v.3, ABI 3130xl) followed by fragment separation on an ABI PRISM 3700 Genetic Analyzer (Thermo Fisher Scientific, Waltham, MA, USA) according to the protocols provided by the manufacturer.

### 2.3. Three-Dimensional Modeling of the INTS1 Protein Mutation Site

The 3D structure of human Integrator Complex Subunit 1 (INTS1) (Uniprot ID: Q8N201) was predicted using the SWISS-MODEL homology-modeling server [13]. As a template, a structure of INTAC-PEC (ID: 7ycx.1.A) was used [14]. The template had 100% sequence identity with the INTS1 protein, as the INTAC-PEC was assembled using purified human INTAC [15]. The particles were reconstructed by means of cryo-electron microscopy (cryo-EM) with a high resolution of 4.2 Å and a high global model quality estimate (GMQE) score of 0.67. Both the global (0.73) and per residue (p.382Gln: 0.83; p399ser: 0.83) QMEANDisCo scores were high. The modeling was performed for the wild-type and mutated proteins: p.Arg382Gln and p.Gly399Ser. The 7ycx.1.A template was further used in mCSM online software [16] in order to predict the protein stability change and the protein–DNA complex affinity change for both examined mutations. The tool employs graph-based signatures to depict surrounding protein residues by encoding the distance patterns between atoms.

## 3. Results

### 3.1. Clinical Report

**Figure 1:**
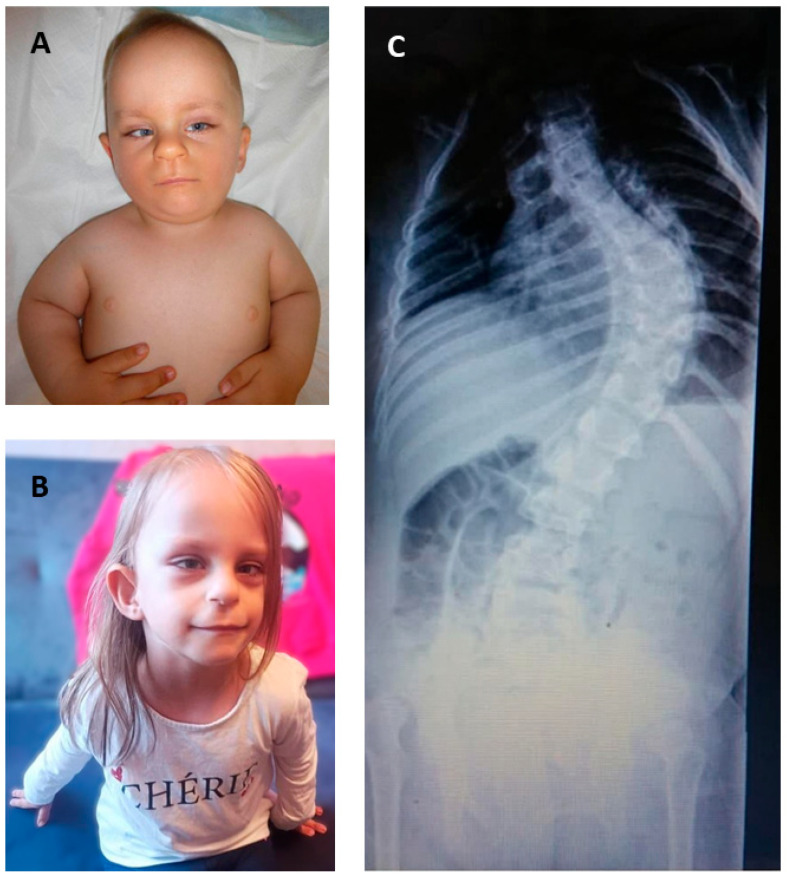
Clinical features observed in the proband. (**A**) Craniofacial dysmorphism at 20 months of age, including broad nasal bridge, bulbous nasal tip, strabismus, deep philtrum, and low-set ears. (**B**) Craniofacial appearance at 5years of age. (**C**) Spinal X-ray showing severe scoliosis.

The proband was a 9-year-old female presenting with psychomotor developmental delay, multiple congenital anomalies, and dysmorphic features. She was born prematurely at 31 weeks of gestation via emergency cesarean section. Her birth weight was 1180 g (10–25th percentile), her length was 39 cm (below the 3rd percentile) [17], and her Apgar scores were 6–7–8–8. She was the first child of a healthy, non-consanguineous couple of Eastern European origin. The prenatal course was complicated by pregnancy-induced hypertension and gestational diabetes (both well controlled), oligohydramnios, and intrauterine growth restriction (IUGR). There was no exposure to known teratogenic factors during pregnancy. A postnatal evaluation revealed several anomalies. Abdominal ultrasound showed left renal agenesis (a horseshoe-shaped right kidney was also identified on follow-up imaging at 3 years of age). Cranial ultrasonography revealed grade I–II intraventricular hemorrhages, and echocardiography identified a patent foramen ovale (PFO). Orthopedic examination revealed bilateral congenital hip dislocation and pes planovalgus deformity. Clinical genetic evaluation at 22 months revealed craniofacial dysmorphism, including frontal bossing with a high frontal hairline, a broad nasal bridge, a long and deep philtrum, a bulbous nasal tip, a thin vermillion border, low-set dysplastic ears, and strabismus. The dysmorphic features observed at 22 months and 5 years of age are shown in Figure 1A,B. Psychomotor development was delayed, with independent walking achieved at 20 months of age. Speech development was also delayed, and behavioral evaluation supported a diagnosis of autism spectrum disorder. Additional findings included short stature, flexion contractures of the knees and elbows, shortening of the long bones, and progressive scoliosis (80°), which required surgical correction (Figure 1C). At the most recent follow-up, the patient also presented with profound visual impairment and was undergoing ophthalmologic evaluation for suspected optic nerve atrophy. The detailed clinical findings of the patient are summarized in Table 1.

### 3.2. Molecular Findings and In Silico Assessment

**Figure 2:**
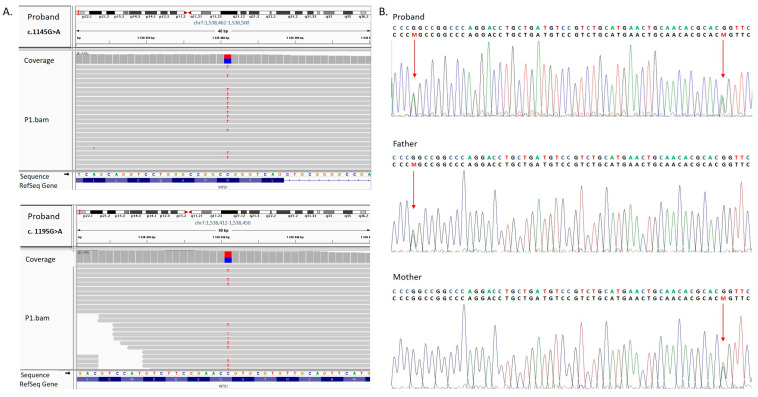
(**A**). The results of whole-exome sequencing visualized using the Integrative Genome Viewer (IGV) tool. The analysis revealed two novel heterozygous variants in *INTS1* (NM_001080453.3): c.1145G>A (p.Arg382Gln) and c.1195G>A (p.Gly399Ser). (**B**). The results of Sanger sequencing showing compound heterozygous *INTS1* (NM_001080453.3) variants identified in the examined family. The proband carries two missense heterozygous variants, c.1145G>A and c.1195 G>A. Variant c.1145G>A was identified in the unaffected father, whereas variant c.1195G>A was identified in the unaffected mother. The variants were depicted by “M” and indicated by an arrow.

**Figure 3:**
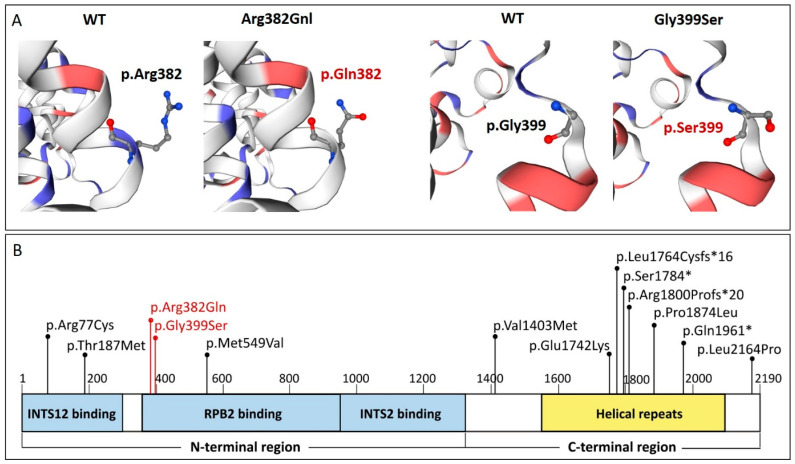
(**A**) 3D prediction of p.Arg382Gln and p.Gly399Ser mutation sites and the corresponding WT loci within the INTS1 protein. Red and blue colors indicate negatively and positively charged amino acids. (**B**). Schematic representation of the INTS1 protein [18]. Blue boxes designate the N–terminal region of INTS1 that interacts with subunits of the Integrator complex (INTS12, INTS2) and PolII subunit RPB2. The yellow box designates helical repeats of the C-terminal region of INTS1. Vertical black and red bars indicate previously reported variants [4,9,10,11] and variants identified in this study, respectively.

Whole-exome sequencing (WES) performed in the proband identified two heterozygous missense variants, NM_001080453.3:c.[1145G>A];[1195G>A] (NP_001073922.2:p.[(Arg382Gln)];[(Gly399Ser)]), in *INTS1*, both located in exon 9 (Figure 2A and Figure 3B). The WES results were confirmed by targeted Sanger sequencing. Segregation analysis showed that the asymptomatic mother carried the c.1195G>A variant, while the asymptomatic father carried the c.1145G>A variant (Figure 2B).

Details of the identified *INTS1* variants are provided in Table 2. Both were very rare, with minor allele frequencies (MAF) of 0.00000138 for c.1145G>A (rs1782994531) and 0.0000248 for c.1195G>A (rs1439392344) according to gnomAD exomes v4.1. Only the latter was detected in gnomAD genomes v4.1 (total allele frequency 0.0000131); the former was absent. Moreover, no homozygotes for either variant were reported in gnomAD v4.

The missense variants c.1145G>A (p.Arg382Gln) and c.1195G>A (p.Gly399Ser) received mostly “pathogenic” and “inconsistent” classifications, respectively, based on consensus in silico predictions generated by multiple computational tools.

The graphical presentation of the p.Arg382Gln mutation site shows a change in net charge at pH 7.4 from positive (p.Arg382) to neutral (p.Gln382). The Three-Dimensional structure of the p.Gly399Ser variant reveals a change from non-polar glycine (Gly) to serine, which contains a polar side chain capable of forming hydrogen bonds (Figure 3A). The evaluation with the mCSM tool identified p.Arg382Gln as stabilizing (0.136 kcal/mol) with regard to protein stability change (ΔΔG) and destabilizing (−0.912 kcal/mol) with regard to protein–DNA complex affinity change. The analysis of p.Gly399Ser predicted the variant to be destabilizing for both protein stability change (−0.616 kcal/mol) and protein–DNA complex affinity change (−0.082 kcal/mol) (Table 2).

According to the guidelines established by the American College of Medical Genetics and Genomics (ACMG), both the c.1145G>A (PP4, PM2,) and c.1195G>A (PP4, PM2, BP4) variants are classified as variants of uncertain significance (VUS) [12] (Table 2). Neither variant has been previously described in the scientific literature. The c.1145G>A variant is absent from ClinVar, whereas the c.1195G>A variant in *INTS1* was reported by another submitter and classified as VUS; however, no phenotypic information was provided. Comprehensive analysis of WES data did not reveal any additional variants, including variants of uncertain clinical significance, that could plausibly explain the patient’s phenotype, nor any potentially pathogenic variants in candidate genes.

## 4. Discussion

In this paper, we report on a 9-year-old female proband carrying two novel compound heterozygous missense variants in *INTS1* (c.1145G>A, p.Arg382Gln; c.1195G>A, p.Gly399Ser) and presenting with developmental delay, multiple congenital anomalies, and distinctive craniofacial dysmorphic features. Unlike all previously reported *INTS1*-related cases, our proband did not present with cataracts, and although speech development was delayed, she ultimately achieved functional verbal communication. This study places our findings in the context of previously published reports, examines genotype–phenotype correlations, and broadens the current spectrum of *INTS1*-related disorders.

To our knowledge, 12 cases with biallelic variants in the *INTS1* gene have been reported in the literature to date. Of these, eight patients originated from four independent families, whereas three unrelated individuals carried identical recurrent variants, resulting in a total of seven distinct variant constellations described thus far. A comprehensive clinical and molecular characterization of all published cases is summarized in Table 1 [4,9,10,11]. Individuals with biallelic *INTS1* variants exhibit a highly heterogeneous phenotypic spectrum encompassing dysmorphic facial characteristics, motor and cognitive delay, skeletal anomalies, ophthalmological findings, and internal organ malformations.

Our proband presented with several of these features, including facial dysmorphism, short stature, and motor delay (all observed in previously reported patients); neurodevelopmental challenges; and certain skeletal problems such as progressive scoliosis. Although she demonstrated delayed speech acquisition, it is noteworthy that she was the only reported patient who developed fluent verbal communication. In contrast to all previously published cases, our proband did not present with cataracts. These distinctions further highlight the phenotypic variability among patients with biallelic *INTS1* variants.

The INTS1 protein is a core component of the Integrator complex and is involved in transcription termination through recruitment of the paused elongation complex (PEC) and cleavage of different RNA polymerase II (Pol II) transcripts [19]. It has recently been shown that the integrator dephosphorylates the C-terminal domain of Pol II as it associates with protein the phosphatase 2A core enzyme (PP2A-AC), forming a complex termed INTAC. The protein’s N-terminal HEAT (huntingtin, elongation factor 3, protein phosphatase 2A, and TOR1) domain forms a tail module of the INTAC complex which is in direct contact with the RPB2 external subunit of Pol II and potentially enables the recruitment of paused elongation complex (PEC). The RPB2 domain interacts with the RNA-DNA hybrid, possibly stabilizing it [14].

In our proband, both variants are situated in a helical domain within the N-terminal region of INTS1 (residues 357 to 1316) that binds to the PolII subunit RPB2 (residues 357 to 948) [18]. The p.Arg382Gln mutation in the INTS1 protein substitutes a positively charged arginine with a polar but uncharged amino acid, glutamine, which may potentially change the electrostatic interactions between these proteins and impair the Integrator complex’s stability. The second variant, p.Gly399Ser, leads to the substitution of highly flexible, non-polar glycine with less flexible serine, which contains a polar side chain with the ability to form hydrogen bonds with other molecules. Thus, this alteration may also affect the structural integrity of the complex by reducing local flexibility and disrupting the protein’s secondary structure

In previously published studies, pathogenic variants in the *INTS1* gene predominantly comprised protein-truncating variants (PTVs; stop-gain, frameshift) or combinations of a missense variant with a truncating variant, most frequently involving the C-terminal region of the protein [4,9,10,11]. Two missense variants located in the C-terminal region (p.Pro1874Leu and p.Leu2164Pro) have also been described, and in silico modeling suggested that these changes could disrupt proper helix folding within this part of the protein [9], thereby potentially impairing the ability of INTS1 to interact with other members of the Integrator complex. By contrast, missense variants affecting the N-terminal region have been exceedingly rare and, to date, have only been reported in two cases, p.Arg77Cys [9] and p.Met549Val [10], both occurring in trans with a truncating variant in the C-terminal region.

In our proband, the two identified missense variants affecting the N-terminal region of the protein represent, to the best of our knowledge, a previously unreported constellation, which may partly explain the milder clinical phenotype observed in the index patient (Figure 3B).

As previously noted, the Integrator complex is a multi-protein assembly, and the precise mechanisms through which these components, including INTS1, interact and coordinate their biological functions remain the subject of ongoing investigations. Zhang et al. [10] performed a broad in silico analysis of co-expression and genetic interaction networks to better understand the phenotypes associated with pathogenic variants in *INTS1*. Their analysis demonstrated that *INTS1* and *INTS8* are functionally interconnected, which may explain the substantial phenotypic overlap observed in patients with pathogenic variants in these genes—an overlap previously highlighted by Oegema et al. [4]. Biallelic pathogenic variants in *INTS8* have been identified as the cause of neurodevelopmental disorder with cerebellar hypoplasia and spasticity (NEDCHS, OMIM #618572), characterized by severe developmental delay, significant intellectual disability, and absence of speech development. In addition, Zhang et al. demonstrated that *INTS1* and *INTS8* are functionally associated with *CTDP1*, a gene whose biallelic pathogenic variants lead to congenital cataracts–facial dysmorphism–neuropathy (CCFDN) syndrome (OMIM #604168). Unlike patients with *INTS1* and *INTS8* variants, individuals with CCFDN usually present with only borderline or mild intellectual disability and are often able to acquire speech [4,10,20].

Our patient has exhibited cognitive impairments, as indicated in Table 1, mainly affecting complex attention, executive function, and social cognition. Interestingly, she has developed speech, shows no intellectual disability, and attends school, features that more closely resemble the profile of CCFDN patients than that of previously reported *INTS1* or *INTS8* cases. Moreover, her profound visual impairment, currently under ophthalmologic evaluation for suspected optic nerve atrophy, is consistent with findings reported in *INTS8* patients by Oegema et al. [4] but not typically observed in *INTS1* cohorts.

In addition to other clinical features previously mentioned, our patient has experienced sleep disturbances characterized by difficulty initiating sleep since early childhood, as reported by her mother. This observation aligns with recent findings by Confino et al. [11], who, using an *Ints1* knockout zebrafish model, demonstrated that Integrator complex dysfunction disrupts circadian rhythms and sleep. The authors suggested that this effect may be partly mediated by altered norepinephrine production in the locus coeruleus, potentially impairing sleep initiation.

Both variants identified in the *INTS1* gene in our patient were classified as VUS. However, taking into account the exclusion of other possible causes, including prenatal exposure to known teratogenic factors, copy number variants, and additional variants in WES data that could plausibly explain the phenotype, we conclude that an *INTS1*-related disorder is the most likely diagnosis in our patient.

The major limitation of this work is the lack of functional studies, which would be highly beneficial for elucidating the effects of the identified variants on INTS1 function but were beyond the scope of this project. The strengths of this study include the detailed clinical characterization of the proband in the context of previously published cases, thus refining genotype–phenotype correlations and likely providing further insights into the clinical presentation of *INTS1*-related disorders. Another limitation is that this study presents the case of a single patient. However, given the very small number of *INTS1*-related cases reported to date, even a single, well-characterized patient may contribute to the understanding of this rare disorder.

In conclusion, our study may expand the genotypic spectrum of *INTS1*-related disease by reporting two previously unreported variants, c.1145G>A p.(Arg382Gln) and c.1195G>A p.(Gly399Ser). Clinically, we describe a patient with a milder phenotype compared to previously reported *INTS1* cases, characterized by the development of fluent and functional speech, the absence of intellectual disability, and the absence of cataracts, thereby broadening the associated phenotypic spectrum. Our findings, in line with previous reports, support the concept of an overlapping clinical spectrum between *INTS1-* and *INTS8*-related disorders. It appears warranted that future studies investigate whether the observed phenotypic variability reflects the impact of individual variants on the global function of the Integrator complex—beyond single subunits—and whether this influence extends to functionally associated genes, such as *CTDP1*, within interconnected pathways. Furthermore, clarifying the extent to which protein–protein interactions and broader functional networks modulate the clinical phenotype remains an important objective.

## Figures and Tables

**Table 1:** Clinical and molecular findings in patients with biallelic INTS1 variants.

−	This Study	Previously Reported Cases												
		Family 1		Family 2		Family 3		−	−	−	−	Family 4		
Features	Our Patient	P2 [10] ^a^	P3 [10]	P4 [9]	P5 [9]	P6 [9]	P7 [9]	P8 [9]	P9 [4]	P10 [4]	P11 [4]	P12 [11]	P13 [11]	Total
Variant: NM_004429.4	c.1145G>A, p. (Arg382Gln); c.1195G>A, p. (Gly399Ser)	c.1645A>G, p. Met549Val; c.5881C>T, p. Gln1961Ter		c.229C>T, p. (Arg77Cys); c.5398dupC, p. (Arg1800ProfsTer20)		c.5621C>T, p. (Pro1874Leu); c.4207G>A, p. (Val1403Met)		c.5290delC, p. (Leu1764CysfsTer16); c.6491T>C, p. (Leu2164Pro)	c.5351C>A,p. (Ser1784Ter),	c.5351C>A,p. (Ser1784Ter)	c.5351C>A,p. (Ser1784Ter)	c.5224G>A,p. (Glu1742Lys); c.560C>T, p. (Thr187Met)		
Zygosity	Het ^b^	Het	Het	Het	Het	Hom ^c^	Hom	Het	Hom	Hom	Hom	Hom	Hom	
Sex	F	F	M	M	M	M	F	M	F	M	M	M	F	
Age at last examination	9 yr	5 yr	11 yr	14 yr	9 yr	11 yr	6 yr	21 mo	10 yr	19 yr	6 yr	9 yr	7 yr	
Short stature	+	+	+	+	+	+	+	+	+	+	+	+	+	13/13
**Facial Dysmorphism**														
Frontal Bossing	+	+	−	−	−	+	+	−	+	+	+	−	−	7/13
High anterior hairline	+	+	−	−	−	−	−	−	+	+	+	−	−	5/13
Horizontal eyebrows	+	−	+	+	+	−	−	−	+	+	+	−	−	7/13
Hypertelorism	−	+	+	+	+	+	+	−	+	+	+	+	+	11/13
Broad nasal bridge	+	+	+	+	+	+	+	−	+	+	+	+	−	11/13
Bulbous nasal tip	+	+	+	+	+	−	−	+	+	+	+	−	−	9/13
Low-set ears	+	−	−	+	+	−	−	−	+	+	+	−	−	6/13
Dysplastic/Prominent ears	+	−	−	+	+	−	−	−	+/−	+	+	−	−	5/13
Deep and long philtrum	+	−	−	+	+	−	−	−	−	−	−	−	−	3/13
Downturned corners of mouth	−	+	−	+	+	+	+	−	+	+	+	−	−	8/13
Abnormal dentition	+	+	ND ^d^	+	+	+	−	−	+	+	+	−	+	8/12
**Neurological and** **Neurodevelopmental**														
Absent speech/severely impaired speech	− *	+	+	+	+	+	+	+	+	+	+	+	+	12/13
Cognitive impairment	+ **	+	+	+	+	+	+	+	+	+	+	+	+	13/13
Motor impairment	+	+	+	+	+	+	+	+	+	+	+	+	+	13/13
Seizure	−	−	−	−	−	−	−	−	+	−	−	+	+	3/13
Hypotonia	+	ND	ND	+	+	+	+	+	+	+	+	+	+	11/11
Autism	+	+	−	−	+	+	+	−	−	−	−	−	−	5/13
**Ocular**														
Cataract	−	+	+	+	+	+	+	+	+	+	+	+	+	12/13
Strabismus	+	+	+	−	−	+	−	−	+	+	+	+	−	9/13
Abnormality of refraction	+	ND	ND	+	−	+	+	−	−	+	+	ND	ND	6/9
**Skeletal**														
Scoliosis	+	ND	ND	−	+	−	−	−	−	−	+	−	+	4/11
Hip dysplasia	+	ND	ND	−	−	−	−	−	−	+	+	−	−	3/11
Pectus abnormality	+	+	−	+	+	−	−	−	+	+	+	−	−	7/13
Overlapping toes	+	ND	ND	+	+	−	−	−	+	+	+	−	−	6/11
**Visceral anomalies**														
Renal malformation	+	ND	ND	−	+	−	−	+	−	+	−	−	−	4/11
Congenital heart disease	+	ND	ND	−	−	−	−	−	−	+	+	+	−	4/11

P = patient; (+) = feature present; (−) = feature absent; ^a^ [ ] = references from the main text; ^b^ Het = heterozygous; ^c^ Hom = homozygous; ^d^ ND = no data; * speech delay was observed; ** cognitive impairment mainly concerned complex attention, executive function, and social cognition.

**Table 2:** The overview and classification of variants found in the *INTS1* gene available from online tools.

	c.1145G>A p.(Arg382Gln)	c.1195G>A p.(Gly399ser)
**gDNA level**	Chr7(GRCh38):g.1498845C>T	Chr7(GRCh38):g1498795C>T
**dbSNP rs number**	rs1782994531	rs1439392344
**Exon**	9	9
**gnomAD Exomes (*v4*)**	Total: 0.00000138; NFE: 0.0000009027	Total: 0.0000248, NFE: 0.0000307
**gnomAD Genomes (*v4*)**	-	Total: 0.0000131, NFE: 0.0000294
**ACMG variant classification**	Uncertain significance	Uncertain significance
**Criteria**	PP4 Supporting PM2 Moderate	PP4 Supporting PM2 Moderate BP4 Moderate
**mCSM: Protein stability change (ΔΔG)**	Stabilizing (0.136 kcal/mol)	Destabilizing (−0.616 kcal/mol)
**mCSM: Protein–DNA affinity change (ΔΔG)**	Destabilizing (−0.912 kcal/mol)	Destabilizing (−0.082 kcal/mol)

Variants are described according to the *INTS1* reference transcript NM_001080453.3 and protein NP_001073922.2. Online tools used: Varsome Premium, mCSM (template used: 7ycx.1.A). Abbreviations: gDNA: genomic DNA; NFE: non-Finnish European.

## Data Availability

Data generated during this study are included in this published article.
